# Does Periodontal Inflammation Affect Type 1 Diabetes in Childhood and Adolescence? A Meta-Analysis

**DOI:** 10.3389/fendo.2020.00278

**Published:** 2020-05-05

**Authors:** Biagio Rapone, Massimo Corsalini, Ilaria Converti, Maria Teresa Loverro, Antonio Gnoni, Paolo Trerotoli, Elisabetta Ferrara

**Affiliations:** ^1^Department of Basic Medical Sciences, Neurosciences and Sense Organs, “Aldo Moro” University of Bari, Bari, Italy; ^2^Interdisciplinary Department of Medicine, “Aldo Moro” University of Bari, Bari, Italy; ^3^Division of Plastic and Reconstructive Surgery, Department of Emergency and Organ Transplantation, “Aldo Moro” University of Bari, Bari, Italy; ^4^Medical Statistic, Department of Biomedical Science and Human Oncology, University of “Aldo Moro” Bari, Bari, Italy; ^5^Complex Operative Unit of Odontostomatology, Hospital S.S. Annunziata, Chieti, Italy

**Keywords:** type 1 diabetes, periodontitis, periodontal disease, children, adolescents

## Abstract

The emergence of link between periodontal disease and diabetes has created conditions for analyzing new interdisciplinary approach making toward tackling oral health and systemic issues. As periodontal disease is a readily modifiable risk factor this association has potential clinical implications. The aim of this paper was systematically review the extant literature related to analytics data in order to identify the association between type 1 diabetes (T1DM) in childhood and adolescence with periodontal inflammation. Following Preferred Reporting Items for Systematic Reviews and Meta-Analyses (PRISMA) guidelines, we conducted a database search between 2004 and 2019. A manual search of the literature was conducted as an additional phase of the search process, with the aim of identifying studies that were missed in the primary search. One hundred and thirty-nine records were screened and 10 fulfilled the inclusion criteria. Most studies were of moderate methodological quality. Outcomes included assessments of diabetes and periodontal status. In diabetic populations, compared to healthy subjects, interindividual differences in periodontal status are reflected in higher severity of periodontal inflammation. The most reported barriers to evidence uptake were the intrinsic limits of cross-sectional report data and relevant research, and lack of timely research output. Based on the evidence presented within the literature, the aforementioned biomarkers correlate with poor periodontal status in type 1 diabetic patients. Whilst the corpus of the evidence suggests that there may be an association between periodontal status and type 1 diabetes, study designs and methodological limitations hinder interpretation of the current research.

## Introduction

According to the literature, the most robust evidence among the association between periodontal disease and systemic disorders (1–7), concerns the biunivocal relationship between periodontal inflammation and diabetes, suggesting a consistent connection with the metabolic control (8–12). Periodontal disease (PD), i.e., plaque-induced inflammation of the periodontium, is an inflammatory disease bacteria-induced that results in the progressive destruction of the attachment apparatus of natural teeth, investing progressively the gingiva, periodontal ligament, and alveolar bone, which originates with the inflammation involving the region of the marginal gingiva above the crest of bone (13–15). Many large-scale prospective studies have shown that periodontitis is associated with elevated inflammatory markers in otherwise healthy populations, demonstrating that the C-reactive protein (CRP) is strongly correlated with PD severity (16–19). The study conducted by Poplawska-Kita et al. (20) underlined the difference between CRP, fibrinogen, and TNF-α levels in periodontics and patients with T1DM compared to subjects without periodontitis and increased levels of systemic inflammation have been reported in relation with poor periodontal status. However, in the same cohort of patients, a good metabolic control was related to lower CRP concentrations, demonstrating a mechanistic link between poor metabolic control and systemic inflammation. Although it was hypothesized that periodontal inflammation could promote the inflammation in systemic compartment, evidence from this study indicated no correlation between periodontitis and circulatory inflammatory markers in type 1 diabetes (T1DM). Indeed, several studies demonstrated that periodontal chronic inflammation is linked to persistent low-grade systemic inflammation (18, 21), concluding that could be involved as a risk factor in the pathogenesis of multiple systemic disorders, including obesity and diabetes mellitus (DM) (14, 15). Particularly, the pathogenic relevance of periodontal inflammation in type 2 diabetes has been amply confirmed in literature, while the nature of connection between T1DM and periodontitis have not yet found a clarification. It has been postulated that the peripheral inflammation might a stimulatory effect on blood glucose concentration. Periodontal treatment was shown to decrease blood glucose level in non-insuline dependent individuals by decreasing of systemic inflammation (22–24). However, despite a plethora of studies concerning the relationship between periodontitis and diabetes, the potential mechanism underlying the potential association between T1DM and periodontal inflammation in childhood remains unclear. Type 1 diabetes is an autoimmune disorder affecting the peripheral system, characterized by a chronic anti-self-inflammatory response (22, 23). The inflammation is a core feature of autoimmune disorder and periodontitis. Increased secretion of pro-inflammatory cytokines, including IL-1, IL-8, IL-6, and TNF-α, during periodontitis development, could exert its effect activating inflammatory pathways in patients with diabetes (25–27). This led to the general hypothesis that this chronic peripheral inflammation could play a key role in exacerbation of systemic inflammatory response that could be a risk factor for the dysregulation of metabolic control in T1DM (28–30). In consideration of the important role that inflammatory processes play in T1DM, and given that periodontitis is associated with a low-grade systemic inflammatory status and the increase of the inflammatory markers in the blood, such as CRP (31), recent works have been conducted to whether periodontal inflammation may influence the metabolic control of T1DM. With this background, the present meta-analysis was performed to analyze the existing evidence regarding the relationship between periodontal status and T1DM in childhood and in adolescence.

## Materials and Methods

### Data Sources and Search Strategy

We carried out a meta-analysis of studies that investigated the association between periodontal inflammation with T1DM in children and adolescents. We followed the quality of reporting of meta-analysis guidelines [the Preferred Reporting Items for Systematic Reviews and Meta-Analyses (PRISMA) statement] for performing and reporting the present meta-analysis. The articles reviewed in this section were systematically searched between 2004 and 2019 with MEDLINE database using the following medical subject headings (MeSH) terms and keywords: “DM, type 1” OR “type 1 DM” OR “type 1 diabetes” AND “periodontal” OR “periodontally” OR “periodontically” OR “periodontics” OR “periodontics” OR “periodontic” OR “periodontitis” OR “periodontitis” OR “periodontitides” AND “periodontal diseases” OR “periodontal” AND “diseases” OR “periodontal diseases” OR “periodontal” AND “disease” OR “periodontal disease” AND “child” OR “child” OR “children” OR “child's” OR “children's” OR “childrens” OR “childs” AND “adolescences” OR “adolescency” OR “adolescent” OR “adolescent” OR “adolescence” OR “adolescents” OR “adolescent's.” A manual search of the literature was conducted as an additional phase of the search process, with the aim of identifying studies that were missed in the primary search. The search was conducted without any restriction on setting or language to retrieve relevant studies.

### Study Selection

A three-step procedure declined the selection and evaluation process of the eligible studies, as follows: (a) first, three reviewers scored the retrieved titles of references independently based on pre-defined criteria; (b) secondly, abstracts were scored by two reviewers independently; (c) subsequently, the full texts of all potentially relevant articles were then reviewed, and studies were included if they met the following criteria:

(1) defined diagnosis criteria for T1DM in children or adolescents;
(2) reported glycated hemoglobin value (HbA1C) level, age at diagnosis of diabetes, and periodontal inflammatory parameters used to assess and monitor the status of periodontal tissues, as follows:
- Plaque index (PI), used in order to record the level and rate of plaque formation on tooth surfaces (32),
- Gingival bleeding index which evaluates the soft periodontal tissues inflammation: Gingival Index, GI or Bleeding on Probing, BoP (33, 34),
- Probing Depth (PD), measuring the sulci of all teeth in order to evaluate the bone loss (35),
- Measurement of Clinical Attachment Level (CAL), indicator of the periodontal support around a tooth (36),
(3) inclusion of a non-exposed group in prospective studies or a control group in retrospective studies. Conference abstracts, reviews, and qualitative studies (i.e., interviews) were discarded, because of the low quality evidence that could possibly bias the research. Disagreements between reviewers will be resolved by a third reviewer.

## Statistical Analysis

The aim of using a meta-analysis procedure for the study was to evaluate findings of past studies that had examined the association between periodontal disease and T1DM. Standard mean difference (SMD) with 95% confidence interval were determined using Der Simonian and Laird random-effects modeling, when there was a significant heterogeneity between studies.

To compare periodontal status of diabetic and control patients, the measure of GI, CAL, PPD, PI e BOP were used. These variables, quantitative were analyzed with a method of meta-analysis for continuous outcome variables, the standardized difference in mean. The SMDs and 95% confidence intervals (CIs) from each study to evaluate the difference in the periodontal parameters between patients with type 1 diabetes and healthy control participants was pooled. A value of SMD > 0 means that the measure is higher in patients with diabetes respect to controls. Overall results were determined under the assumption of random effects model and were summarized SMD and 95% CI. Because the studies differed in terms of patients, outcome definitions, and study design, a test for heterogeneity of the effect on outcome between the included studies was assessed. The between-study statistical heterogeneity was detected measuring the Cochran's *Q* homogeneity test statistic and the *I*^2^ (inconsistency) index and its associated 95% confidence interval (37). Statistical analyses were performed using MedCalc software, version 19.1.5 (Ostend, Belgium; https://www.medcalc.org; 2020).

### Literature Search

**Figure 1:**
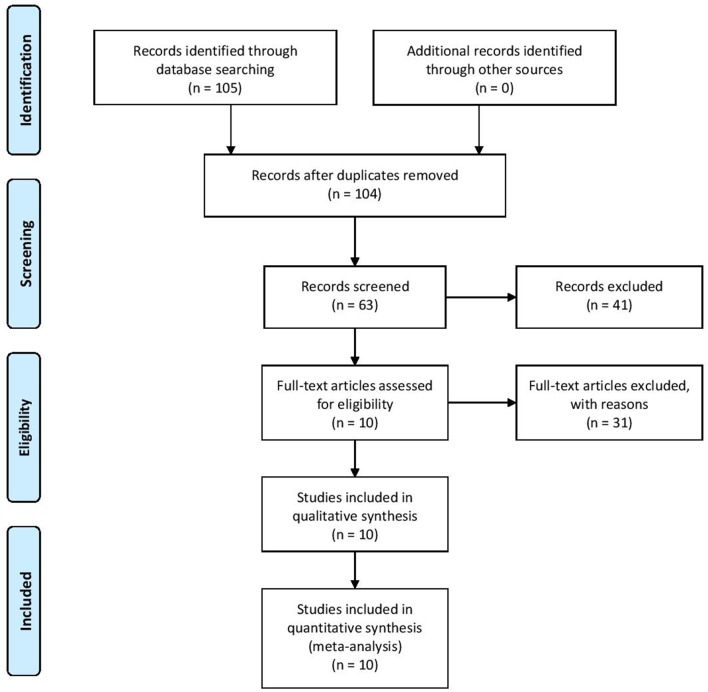
Flow diagram of study selection.

Overall, 105 references were initially identified. After the initial screening of titles and abstracts, a total of 63 articles were discarded, leaving 41 articles for retrieval. Full text assessment of these articles resulted in 10 eligible articles that met our inclusion criteria (38–46). Figure 1 displays the process of selection studies.

### Study Characteristics and Statistical Analysis

Table 1 shows the main characteristics of the included articles. The examined papers were quite heterogeneous. Seven were comparative-cross sectional studies (37–43) and three were observational case-control studies (40–42). The method of outcomes ascertainment varied across articles. The studies focused on the following indices of periodontal inflammation, including the afore mentioned Plaque Index (PI), Gingival Index (GI), Bleeding on Probing (BoP), Probing Pocket Depth (PPD), and Clinical attachment level (CAL).

**Table 1:** Characteristic of the sample in the selected studies.

**Study**	**Type of study**	**No. of diabetic patients**	**No. of controls**	**Duration of diabetes mean (SD)**	**Age of diabetic mean (ds)**	**Age of controls mean (ds)**
Lalla et al. (42)	Case-control	182	160	4.5 (8)	11.9 (3.3)	10.9 (2.6)
Columbia						
Dakovic et al. (41)	Comparative, cross-sectional	187	178	5.46 (3.48)		
Serbia						
Al-Khabbaz et al. (38)	Comparative, cross-sectional	95	61	9.7 (2.3)	9.1 (3.9)	8.9 (2.2)
Kuwait						
Rafatjou et al. (43)	Case-control	80	80	5.46 (3.48)	12.5 (4.05)	12.08 (3.47)
Iran						
Ismail et al. (46)	Comparative, cross-sectional	32	32		12 (4)	12 (4)
Hong Kong						
Babu et al. (47)	Comparative, cross-sectional	80	80			
India						
Coelho et al. (40)	Comparative, cross-sectional	36	36	5.67 (3.96)	13	13
Geetha et al. (44)	Case-control	175	175			
India						
Duque et al. (45)	Comparative, cross-sectional	24	27		9.45 (1.69)	9.62 (1.86)
Orbak et al. (39)	Comparative, cross-sectional	50	50		9 (0.14)	9
Turkey						

**Figure 2:**
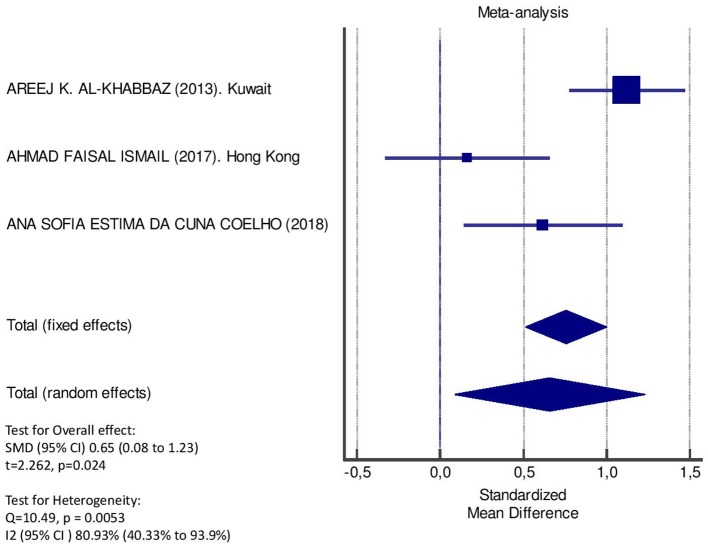
Forest plot of study effects for BOP. In the bottom, the overall effect for random effects model and heterogeneity test.

**Figure 3:**
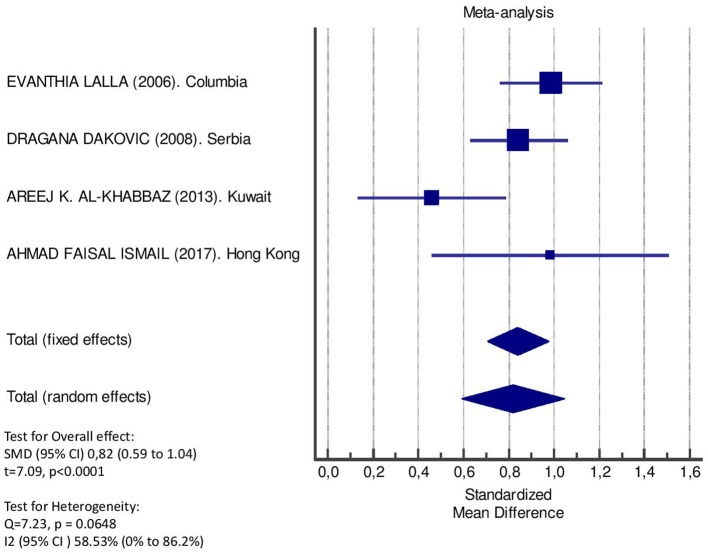
Forest plot of study effects for CAL. In the bottom, the overall effect for random effects model and heterogeneity test.

**Figure 4:**
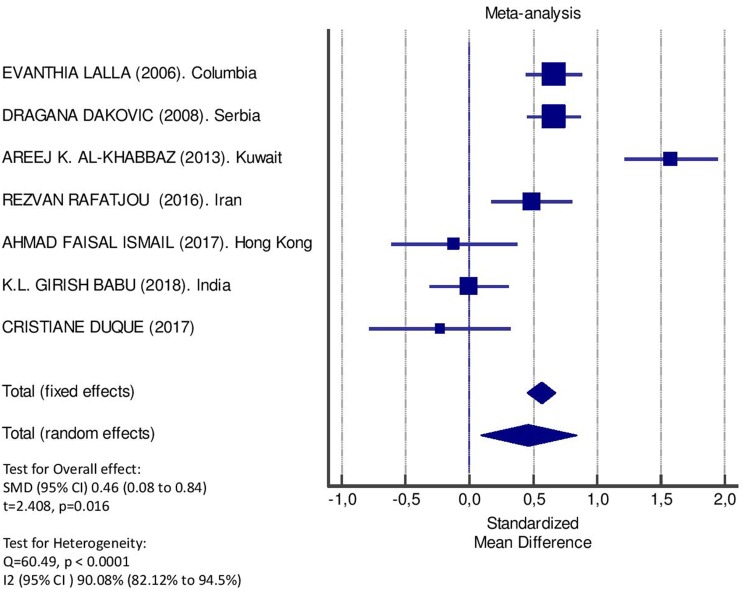
Forest plot of study effects for GI. In the bottom, the overall effect for random effects model and heterogeneity test.

**Figure 5:**
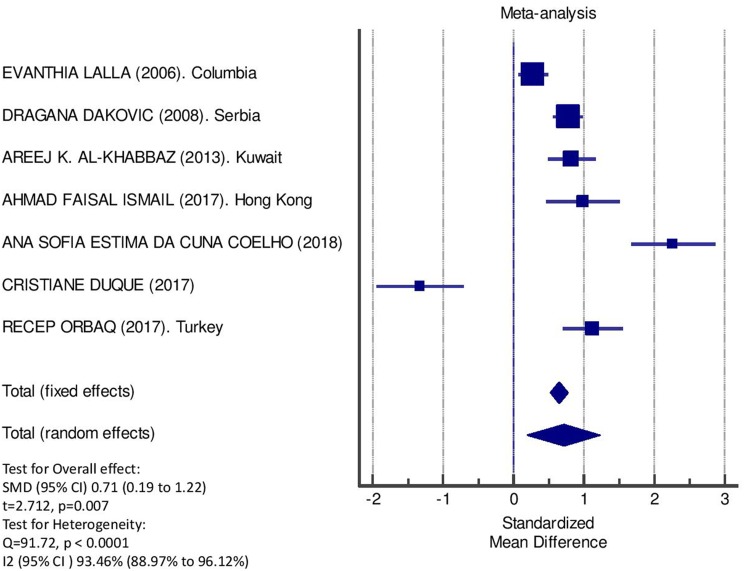
Forest plot of study effects for PI. In the bottom the overall effect for random effects model and heterogeneity test.

**Figure 6:**
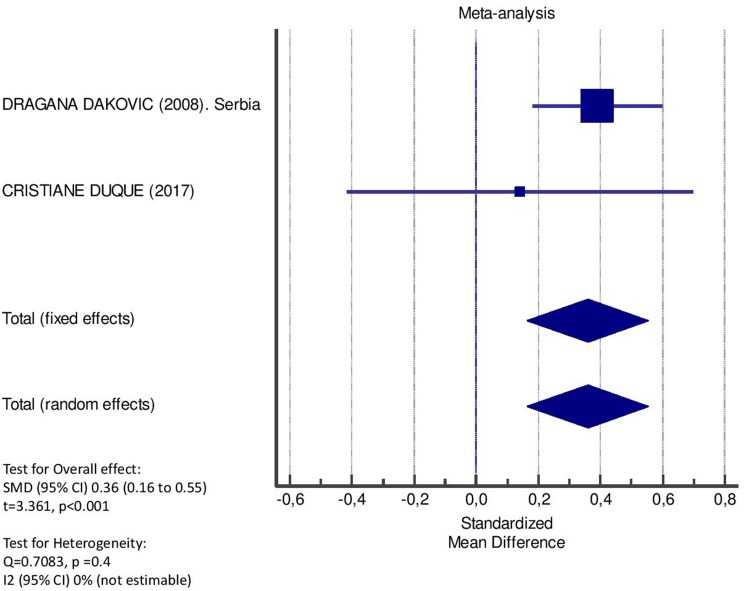
Forest plot of study effects for PPD. In the bottom, the overall effect for random effects model and heterogeneity test.

Table 2 displays the results of the meta-analysis of the 10 articles. The Bleeding on Probing (BOP) was evaluated (Figure 2, Table 2) only in three studies, with a number of diabetic patients ranging from 32 to 95 and controls ranging from 32 to 61. Evidence from the study conducted by Ismail (10) suggested no statistically significant difference between group of patients with diabetes and controls (SMD = −016; −0.33 to 0.66; 95% CI). The other two studies, on the contrary, have shown a statistically significant difference between subjects with diabetes and control groups (11, 12). The Overall SMD (95% CI) was −0.65 (0.09–1.22), that suggests a higher BOP in diabetic respect to controls (*t* = 2.26, *p* = 0.024). The heterogeneity was statistically significant (*Q* = 10.48, *p* = 0.0053) and *I*^2^ was 80.9% (40.3–93.9%). The CAL index was evaluated in four studies (Figure 3, Table 3). The range of the sample size of the studies was 32–187 for group with diabetes and 32–178 in the control group. The SMD was 0.56 (0.13–0.79) in the study from Al-Khabbaz, that was the lower difference, while in the studies by Lalla and Ismail SMD was, respectively, 0.99 (0.76–1.21) and 0.98 (0.46–1.51). The overall value of the difference of CAL between individuals with diabetes and controls was 0.82 (0.59–1.04) and it was statistically significant (*t* = 7.09, *p* < 0.0001). The Cochran's *Q* was 7.2 (*p* = 0.0648) and *I*^2^ was 80.9% (0–86%). Seven studies (Figure 4, Table 4) allowed the evaluation of the GI. The study of Duque was the one with the low sample size, it had 24 patients with diabetes and 27 controls and the SMD was −0.23 with 95% CI: −0.79 to 0.32, results that suggest no statistically significant difference between the two groups. The study by Ismail resulted with no statistically significant difference between the two groups too. The SMD was −012 and its 95% CI was −0.62 to 0.37. The study of Babu, which included 160 patients, 80 individuals for each group, was inconclusive with a SMD = −0 and 95% CI: −0.31 to 0.31. Among the seven studies, only the study conducted by Al-Khabbaz, which compared 95 patients with diabetes vs. 61 controls, showed the higher difference. In this study, SMD was 1.58 (1.21–1.95). The overall SMD resulted 0.46 (0.09–0.84), revealing a statistically significant difference between diabetic and controls (*t* = 2.408, *p* = 0.016). Heterogeneity resulted statistically significant (*q* = 60.49, *p* < 0.0001) and *I*^2^ was 90.1% (82.1–94.5%). The evaluation of PI (Figure 5, Table 5) was made across seven studies. Only the study of Duque (24 diabetics and 27 controls) had a SMD −1.33 (−1.9 to −0.72), suggesting a statistically significant higher value of PI in controls. The other six studies had SMD that underlined a higher value of PI in group of patients with diabetes. The study of Da Cuna Coelho, 72 patients (36 for each group), had the highest value of SMD = 2.27 (1.67–2.87). The study of Orbaq, that involved 100 patients (50 by group) had a SMD of 1.13 (0.7–1.55). The lower value of SMD was that of Lalla: 0.28 (0.07–0.49). The overall SMD was 0.71 (0.19–1.22) and the difference showed a statistically significant higher value of PI in diabetic compared to controls (*t* = 2.71, *p* = 0.007). The heterogeneity was statistically significant (*Q* = 91.72, *p* < 0.0001) and *I*^2^ was 93.5% (88.9–96.1%). Only two studies evaluated the PPD index (Figure 6, Table 6): Duque's study has a 95% CI that suggest non-statistical significance (SMD = 0.14, 95% CI: −0.41 to 0.69); Dakovic's study had a SMD 0.39 (0.18–0.59). The overall SMD was 0.36 (0.16–0.55), showing a statistically significant difference between groups (*t* = 3.63, *p* < 0.001) and patients with diabetes with higher value of PPD. *I*^2^ was not estimable and heterogeneity resulted non-statistically significant (*Q* = 0.708, *p* = 0.4). In general, the association between periodontal disease and type 1 diabetes tended to be positive regardless of the severity of periodontitis and metabolic control degree.

**Table 2:** Main results from the studies reporting BOP and evaluation of standardized mean difference for meta-analysis.

**Study**	**Diabetic**		**Controls**		**Standardized mean difference**	**95% Confidence interval**
	***n*. pts**.	**Mean** **(ds)**	***n*. pts**.	**Mean** **(ds)**		
Lalla et al. (42)						
Columbia						
Dakovic et al. (41)						
Serbia						
Al-Khabbaz et al. (38)	95	0.4 (0.3)	61	0.1 (0.2)	1.12	0.78 to 1.47
Kuwait						
Rafatjou et al. (43)						
Iran						
Ismail et al. (46)	32	0.2 (0.18)	32	0.16 (0.29)	0.16	−0.33 to 0.66
Hong Kong						
Babu et al. (47)						
India						
Coelho et al. (40)	36	35.66 (16.06)	36	26.3 (13.88)	0.62	0.14 to 1.09
Geetha et al. (44)						
India						
Duque et al. (45)						
Orbak et al. (39)						
Turkey						

**Table 3:** Main results from the studies reporting CAL, and evaluation of standardized mean difference for meta-analysis.

**Study**	**Diabetic**		**Controls**		**Standardized mean difference**	**95% Confidence interval**
	***n*. pts**.	**Mean** **(ds)**	***n*. pts**.	**Mean** **(ds)**		
Lalla et al. (42)	182	1.8 (1.1)	160	0.8 (0.9)	0.99	0.76 to 1.21
Columbia						
Dakovic et al. (41)	187	1.1 (0.4)	178	0.8 (0.3)	0.84	0.63 to 1.06
Serbia						
Al-Khabbaz et al. (38)	95	2.6 (0.5)	61	2.4 (0.3)	0.46	0.13 to 0.79
Kuwait						
Rafatjou et al. (43)						
Iran						
Ismail et al. (46)	32	1.8 (1.1)	32	0.8 (0.9)	0.98	0.46 to 1.51
Hong Kong						
Babu et al. (47)						
India						
Coelho et al. (40)						
Geetha et al. (44)						
India						
Duque et al. (45)						
Orbak et al. (39)						
Turkey						

**Table 4:** Main results from the studies reporting GI, and evaluation of standardized mean difference for meta-analysis.

**Study**	**Diabetic**		**Controls**		**Standardized mean difference**	**95% Confidence interval**
	***n*. pts**.	**Mean** **(ds)**	***n*. pts**.	**Mean** **(ds)**		
Lalla et al. (42)	182	1.2 (0.3)	160	1 (0.3)	0.67	0.45 to 0.88
Columbia						
Dakovic et al. (41)	187	0.7 (0.3)	178	0.5 (0.3)	0.67	0.45 to 0.88
Serbia						
Al-Khabbaz et al. (38)	95	1.9 (0.7)	61	0.9 (0.5)	1.58	1.23 to 1.95
Kuwait						
Rafatjou et al. (43)	80	0.45 (0.49)	80	0.26 (0.24)	0.49	0.18 to 0.81
Iran						
Ismail et al. (46)	32	0.58 (0.36)	32	0.62 (0.29)	−0.12	−0.62 to 0.37
Hong Kong						
Babu et al. (47)	80	0.33 (0.48)	80	0.33 (0.53)	0	−0.31 to 0.31
India						
Coelho et al. (40)						
Geetha et al. (44)						
India						
Duque et al. (45)	24	17.6 (8.33)	27	19.1 (3.9)	−0.23	−0.79 to 0.33
Orbak et al. (39)						
Turkey						

**Table 5:** Main results from the studies reporting PI, and evaluation of standardized mean difference for meta-analysis.

**Study**	**Diabetic**		**Controls**		**Standardized mean difference**	**95% Confidence interval**
	***n*. pts**.	**Mean** **(ds)**	***n*. pts**.	**Mean** **(ds)**		
Lalla et al. (42)	182	1.2 (0.4)	160	1.1 (0.3)	0.28	0.07 to 0.49
Columbia						
Dakovic et al. (41)	187	0.9 (0.3)	178	0.7 (0.2)	0.78	0.57 to 0.99
Serbia						
Al-Khabbaz et al. (38)	95	1.8 (0.7)	61	1.3 (0.4)	0.83	0.49 to 1.16
Kuwait						
Rafatjou et al. (43)						
Iran						
Ismail et al. (46)	32	0.76 (0.4)	32	0.46 (0.14)	0.99	0.46 to 1.51
Hong Kong						
Babu et al. (47)						
India						
Coelho et al. (40)	36	52.03 (2.04)	36	38.25 (8.26)	2.27	1.67 to 2.86
Geetha et al. (44)						
India						
Duque et al. (45)	24	24.7 (4.62)	27	32 (6)	−1.33	−1.95 to −0.72
Orbak et al. (39)	50	1.69 (0.4)	50	1.18 (0.5)	1.13	0.7 to 1.55
Turkey						

**Table 6:** Main results from the studies reporting PPD, and evaluation of standardized mean difference for meta-analysis.

**Study**	**Diabetic**		**Controls**		**Standardized mean difference**	**95% Confidence interval**
	***n*. pts**.	**Mean** **(ds)**	***n*. pts**.	**Mean** **(ds)**		
Lalla et al. (42)						
Columbia						
Dakovic et al. (41)	187	1.5 (0.3)	178	1.4 (0.2)	0.39	0.18 to 0.59
Serbia						
Al-Khabbaz et al. (38)						
Kuwait						
Rafatjou et al. (43)						
Iran						
Ismail et al. (46)						
Hong Kong						
Babu et al. (47)						
India						
Coelho et al. (40)						
Geetha et al. (44)						
India						
Duque et al. (45)	24	1.48 (0.48)	27	1.41 (0.5)	0.14	−0.41 to 0.69
Orbak et al. (39)						
Turkey						

## Discussion

Type 1 diabetes is one of the major autoimmune disease developing in childhood, with the incidence rate ranging between 3 and 5% every year (47–49). Periodontitis is a biological process leading to the disruption of the normal physiology of the alveolar bone homeostasis in response to the invasion of pathogenic microorganisms in the periodontium. PD is related to diabetes, and there is an increasing interest in research regarding the effects of periodontal inflammation on children and adolescents with T1DM. The literature demonstrates that inflammatory mediators released during periodontitis development could influence the metabolic control on patients with diabetes. Although PD has been well described in type 2 diabetes (T2DM) as the sixth complication of diabetes (50), there is a paucity of reports regarding the impact of periodontal inflammation on the pathogenesis and progression of T1DM in childhood and adolescence. The pathogenesis of T1DM and T2DM is quite different, probably leading to different risk of PD (51). Studies examining the association of poor periodontal status with glycemic control in subjects diagnosed with T1DM, emphasized on the prevalence of periodontitis in adults (21, 52). It has been observed that patients with diabetes exhibited greater susceptibility for PD. The relationship occurring at a cross-level with T1DM is not well-clarified as that occurring within the T2DM. Despite the quite differences between the pathogenic mechanism in T1DM and T2DM, periodontal implications share many similarities. In this regard, it has been demonstrated that despite the overlap that exists with respect the causes of the two, the increased inflammatory activity has been reported as a common denominator underlying hyperglycemia-induced insulin resistance and diabetic complications. The link between periodontal inflammation and diabetes is sufficiently strong to have prompted the suggestion that the presence of periodontitis ought to be viewed as a key risk factor for pathogenesis of diabetes. This involves promotion of proinflammatory cytokines release. Few evidences indicate that the periodontitis may be involved in the pathogenesis of T1DM, and the data available concerning the potential impact of periodontal inflammation on T1DM in childhood and adolescence is still limited. The data derived from human studies have revealed that the periodontitis chronicity elicits cytokine sensitivity, contributing to the vulnerability to long-term complications. Hyperglycemia is accompanied by an up-regulated expression of TNF-α, interleukin (IL)-1β, IL-6, and IL-18, which contribute to insulin resistance by both JNK and the IKKβ/NF-kβ pathway. It has been shown that periodontitis contributes to the development of low-grade systemic inflammation (53). Oxidative stress is one of the important examples of an essential host factor that can explain the prevalence and severity of periodontitis in patients with diabetes. The activity of the prooxidative enzyme myeloperoxidase in the gingival crevicular fluid of periodontal patients with diabetes is reduced compared to non-diabetics and non-periodontal patients with diabetes (54, 55). Parodontotic patients with diabetes shown an imbalance between the prooxidants and antioxidants in the body, thus promoting cellular injury and increasing the magnitude of inflammatory response effects. This leads to the question of how periodontal abnormalities and the related inflammatory changes are able to impact on the pathogenesis of T1DM in children and adolescents. Patients with diabetes are a greater risk for PD than healthy individuals but it didn't seem to be any impact for T1DM development. Our systematic analysis and meta-analysis persuasively explain that the severity of periodontal inflammation is increased in children and adolescents with T1DM compared to that in healthy controls. We systematically reviewed the published literature to evaluate the relationship between metabolic control in children and adolescents affected by T1DM and PD, but the great important set of studies into the impact of different kinds of systemic inflammation has been carried focusing on the adult patients or heterogeneous groups as the primary interest for consideration (49, 50). Our results are consistent with those reported by Löe (51), who conducted a systematic review and meta-analysis to investigate the association between T1DM and T2DM mellitus and PD. Interestingly, they have shown that the overall difference in the average gingival index between individuals with and without diabetes increased significantly. Roy et al. (52) also demonstrated that, when patients with diabetes were compared to non-diabetics in regard to clinical attachment loss, the pooled difference increased more in patients with T2DM (0.652) (95% CI: 0.465–0.840; Pb.0001) than in subjects with T1DM (0.691) (95% CI: 0.427–0.956; Pb.0001). The strong interindividual variability in oral health status depends on a number of factors, both constitutional and environmental, that determine the different clinical cases developmental. The research conducted by Listgarten et al. (35) revealed that the age impacted negatively on increased gingival inflammation, but also more accumulation of bacterial plaque and calculus deposits, the key pathogenic factors of periodontitis, were scored in children with diabetes than in the healthy groups. The causes of this process appear to be related to vascular changes which are part of the pathogenesis of diabetes complications, which may involve also the periodontal vasculature (52). The alterations of vascular components may result in increased gingival bleeding. Furthermore, the significant reduction of the salivary secretion rate it has been documented in children with T1DM when compared to healthy children (55). Similar findings have also been reported in another studies, which performed analysis for all periodontal parameters, showing that the gingival index, bleeding on probing, and higher dental plaque amount were significantly associated with T1DM in children (35–37, 39). A poor association was reported in patients with diabetes with periodontitis; however, this finding could not explain the association between diabetes and periodontitis in children and adolescents (52). A major finding of our review was that the periodontal status (PI and GI parameters) affects the impacts of uncontrolled diabetes (56), and this has practical implications for multidisciplinary management of primitive pathology. A similar conclusion was previously reached by a systematic review on the impact of periodontitis on T1DM (57). The emergence of close link between periodontal disease and diabetes has created conditions for analyzing a new interdisciplinary approach making toward tackling oral health and systemic issues.

### Limitations

The major limitation of the mentioned studies was the inexpensive nature of cross-sectional study design. Another restriction was the paucity and often low quality of primary data. The paucity of studies likely reflects the fact that periodontitis has not been identified as a serious issue in T1DM, because of the natural history of disease. However, there is no doubt that children with diabetes have poor oral hygiene. Another major limitation of the evidence base was the absence of primary research on the effectiveness of periodontal treatment to affect glycemic control in T1DM; this was particularly notable in comparison with the abundance of studies on T2DM. Due to this limitation, we were only able to analyze the overall correlation between periodontal parameters.

Study selection, data extraction, and quality assessment were performed independently by two authors according to predesigned criteria to minimize bias and transcription errors. Potential limitations of this meta-analysis arise from the unavailability of individual participant data from the included studies. Our meta-analysis was based on studies that varied in many ways (study design, population sample, adjustment for confounders, and different ascertainment methods for exposure and outcome), which may be considered another limitation. However, we adopted appropriate meta-analytic techniques with random-effect models, which enabled us to account for these differences. Second, we could not conduct a multivariable meta-regression, due to the small number of studies. Finally, the most reported barriers to evidence uptake were the intrinsic limits of cross-sectional report data and relevant research, and lack of timely research output. Based on the evidence presented within the literature, the poor metabolic control correlate with scarce periodontal status in patients with T1DM.

## Conclusions

Whilst focusing emerging studies concerning the relationship between periodontal disease and type 2 diabetes in health sciences are being implemented in research, the studies of potential effects of periodontal inflammation on children and adolescents affected by T1DM are incomplete. Despite this recent exponential increase in the number of studies implicated in the association between periodontitis and diabetes, no general consensus has yet emerged of a causal effect of periodontal inflammation in T1DM. We have a mosaic of data from diverse results amenable for errors. Instead, we have a mosaic of data from diverse results amenable for errors, of disease interactions. The underlying mechanisms that support the biological connection between periodontitis and T1DM in childhood and adolescence can be discussed in a range of levels: the common implication of inflammation in the pathogenesis of both diseases, the transitory bacteremia originated by periodontal diseases, and the systemic immune inflammation as a response to chronic peripheral infection. In conclusion, this meta-analysis did not provide strong evidence that periodontitis is a significant risk factor for T1DM, and the link between PD and T1DM appears to be not solid as the connection with T2DM, although this association is less clarified. Given the high prevalence and incidence of T1DM in the general population, the observed relationship between PD and diabetes has clinical and public health importance. Further research to identify the link between T1DM and PD is warranted. Available reports evidently suggest a link between the mechanism of periodontal diseases and systemic/metabolic diseases where both conditions could aggravate each other. Despite this recent exponential increase in the number of studies implicated in relationship between periodontitis and T2DM, no general consensus has yet emerged of a causal effect of periodontal inflammation on T1DM. Longitudinal studies and additional research with larger sample sizes are needed to determine whether periodontitis is the result of T1DM or contributes to the worsening of metabolic control in children and adolescents with T1DM.

## Data Availability Statement

All datasets generated for this study are included in the article/supplementary material.

## Author Contributions

Conceptualization: BR; investigation: BR and EF; resources: IC, PT, and EF; data curation: BR, PT, and EF; validation: BR, EF, MC, IC, ML, and AG; writing-original draft preparation, writing-review, and editing: BR and EF; visualization: AG, IC, MC, and PT; supervision: EF and BR; project administration: BR.

## Conflict of Interest

The authors declare that the research was conducted in the absence of any commercial or financial relationships that could be construed as a potential conflict of interest.
